# The diagnosis of tuberculous meningitis: advancements in new technologies and machine learning algorithms

**DOI:** 10.3389/fmicb.2023.1290746

**Published:** 2023-10-24

**Authors:** Yi Shi, Chengxi Zhang, Shuo Pan, Yi Chen, Xingguo Miao, Guoqiang He, Yanchan Wu, Hui Ye, Chujun Weng, Huanhuan Zhang, Wenya Zhou, Xiaojie Yang, Chenglong Liang, Dong Chen, Liang Hong, Feifei Su

**Affiliations:** ^1^Department of Infectious Diseases, Wenzhou Central Hospital, Wenzhou, China; ^2^The First School of Medicine, Wenzhou Medical University, Wenzhou, China; ^3^School of Materials Science and Engineering, Shandong Jianzhu University, Jinan, China; ^4^Department of Infectious Diseases, Wenzhou Sixth People’s Hospital, Wenzhou, China; ^5^Wenzhou Key Laboratory of Diagnosis and Treatment of Emerging and Recurrent Infectious Diseases, Wenzhou, China; ^6^Postgraduate Training Base Alliance of Wenzhou Medical University, Wenzhou, China; ^7^Wenzhou Institute, University of Chinese Academy of Sciences, Wenzhou, China; ^8^School of Electrical and Information Engineering, Quzhou University, Quzhou, China; ^9^The Fourth Affiliated Hospital Zhejiang University School of Medicine, Yiwu, China; ^10^School and Hospital of Stomatology, Wenzhou Medical University, Wenzhou, China; ^11^Wenzhou Medical University Renji College, Wenzhou, China; ^12^Wenzhou Central Blood Station, Wenzhou, China; ^13^Department of Infectious Diseases, The Third Affiliated Hospital of Wenzhou Medical University, Wenzhou, China

**Keywords:** tuberculous meningitis, machine learning, next-generation sequencing, diagnosis, infectious diseases, mycobacterium tuberculosis

## Abstract

Tuberculous meningitis (TBM) poses a diagnostic challenge, particularly impacting vulnerable populations such as infants and those with untreated HIV. Given the diagnostic intricacies of TBM, there’s a pressing need for rapid and reliable diagnostic tools. This review scrutinizes the efficacy of up-and-coming technologies like machine learning in transforming TBM diagnostics and management. Advanced diagnostic technologies like targeted gene sequencing, real-time polymerase chain reaction (RT-PCR), miRNA assays, and metagenomic next-generation sequencing (mNGS) offer promising avenues for early TBM detection. The capabilities of these technologies are further augmented when paired with mass spectrometry, metabolomics, and proteomics, enriching the pool of disease-specific biomarkers. Machine learning algorithms, adept at sifting through voluminous datasets like medical imaging, genomic profiles, and patient histories, are increasingly revealing nuanced disease pathways, thereby elevating diagnostic accuracy and guiding treatment strategies. While these burgeoning technologies offer hope for more precise TBM diagnosis, hurdles remain in terms of their clinical implementation. Future endeavors should zero in on the validation of these tools through prospective studies, critically evaluating their limitations, and outlining protocols for seamless incorporation into established healthcare frameworks. Through this review, we aim to present an exhaustive snapshot of emerging diagnostic modalities in TBM, the current standing of machine learning in meningitis diagnostics, and the challenges and future prospects of converging these domains.

## 1. Introduction

Tuberculosis, caused by mycobacterium tuberculosis, represents one of the major global public health issues (World Health Organization [WHO], 2022). Although it primarily infects the lungs, known as pulmonary tuberculosis (PTB), it can also affect extrapulmonary sites such as the central nervous system (Ohene et al., 2019). Specifically, TBM is a lethal form of tuberculosis, especially among infants and untreated HIV-infected individuals (Heemskerk et al., 2011; Seddon et al., 2019). Despite TBM accounting for only 1% of new diagnoses, its consequences are severe, leading to death or disability in nearly half of the patients (Marais et al., 2010).

Mycobacterium tuberculosis, the causative agent for tuberculosis, is characterized by slow growth and acid resistance, features attributed to its complex cell wall that confer survival advantages within host organisms (Gao et al., 2003). The pervasive transmission of the pathogen, coupled with the intrinsic difficulties associated with bacterial culturing, elevates tuberculosis to a pressing issue in global healthcare. More specifically, these biological complexities present substantial obstacles in the accurate diagnosis and effective management of TBM. While the emergence of drug resistance exacerbates the complexity of treatment regimens, timely diagnosis and intervention can substantially reduce mortality rates (World Health Organization [WHO], 2022). However, early diagnosis of TBM is rendered exceptionally challenging due to the low sensitivity of current diagnostic gold standards and prolonged culture times (Pormohammad et al., 2019). Many patients only seek medical intervention at advanced stages, such as during a mental health crisis or a comatose state (Yan et al., 2020). Typically, TBM diagnosis is predicated on clinical manifestations and empirical treatment rather than concrete diagnostic evidence (Thwaites et al., 2000; Ssebambulidde et al., 2022). Alarmingly, commonly employed clinical markers lack specificity, thereby increasing the risk of misdiagnosing TBM as other types of meningitis, such as viral or bacterial forms (Venkatesan et al., 2013; Wang et al., 2019; Xing et al., 2020; He et al., 2023).

Even in the face of such challenges, emerging technologies signal a positive shift in the diagnostic paradigms for TBM. The application of targeted gene sequencing is increasingly vital for discerning drug-resistant forms of mycobacterium tuberculosis, an essential step for tailoring effective treatment regimens (Feuerriegel et al., 2021). Molecular diagnostic methodologies, such as RT-PCR and miRNA assays, provide sensitive and specific tools for the early diagnosis of TBM, allowing for the rapid identification of both tuberculosis and rifampicin resistance (Nhu et al., 2014; Hu et al., 2019). Moreover, mNGS is gaining traction as a diagnostic tool, with its capacity for the unbiased detection of a diverse array of pathogenic organisms, thereby revolutionizing the field of infectious disease diagnosis (Lin et al., 2023). Through the incorporation of high-throughput mass spectrometry, as well as metabolomic and proteomic analyses, the research landscape for TBM is expanding to include the identification of specific biomarkers, such as metabolites and proteins, that may be intricately linked with the pathophysiology of the disease (Mason and Solomons, 2021). As a result, these technological advancements are significantly enhancing diagnostic precision and facilitating unprecedented monitoring of disease progression. Moreover, MRI provides distinct advantages, particularly in the assessment of cerebral structural alterations and inflammatory responses (Dian et al., 2020).

Machine learning offers a potential solution for improving the diagnostic processes of TBM and other forms of tuberculosis. Machine learning is a computational framework designed to make predictions or decisions by automatically extracting and decoding complex data patterns (Camacho et al., 2018). Specifically, these algorithms utilize feature vectors and corresponding labels to adjust the internal parameters of the model, leveraging a variety of optimization techniques (Capobianco and Dominietto, 2020). By analyzing large volumes of medical images, genomic data, or clinical records, machine learning models can identify complex patterns of disease that may be imperceptible to human experts (Greener et al., 2022). This technology has potential advantages in terms of accuracy and speed and has been widely applied in the field of infectious diseases to improve the diagnosis, detection of complications, treatment, and prognostic stratification (Peiffer-Smadja et al., 2020). Given its track record in related domains, the integration of machine learning into TBM research can thus be both possible and advantageous. Our detailed feasibility analysis, considering the substantial data volume, complexity, and interdisciplinary nature of TBM research, further underscores the significant potential of machine learning techniques. Moreover, by presenting instances where machine learning has been implemented in TBM studies, we emphasize its practical viability and the tangible benefits it brings to the field.

This review aims to provide an overview of new diagnostic technologies for TBM, the current status and progress of machine learning in meningitis diagnosis, and the challenges and future directions when integrating these realms.

## 2. New diagnostic technologies for TBM

**FIGURE 1:**
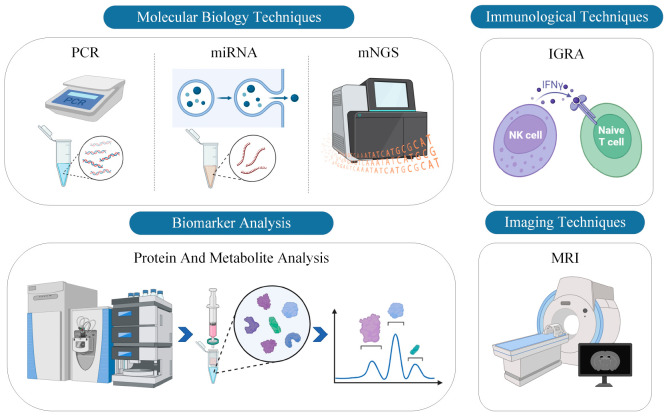
Comprehensive schematic illustration of current diagnostic technologies for TBM. The figure categorizes the diagnostic modalities into four primary technological approaches: PCR, miRNA, mNGS, IGRA, protein and metabolite analysis, and MRI. Each technology is represented with corresponding icons or graphical elements to delineate its unique contribution to the diagnosis of TBM.

Diagnosing TBM requires a multi-faceted approach, employing various advanced technologies and methodologies. Over the years, advancements in molecular biology, immunology, biomarker analysis, and imaging technologies have greatly enhanced our ability to detect and study TBM, providing clinicians and researchers with a broader toolkit for diagnosis and assessment. Each technology, from molecular tools like PCR to imaging modalities like MRI, has its unique advantages and challenges. This section delves into these various diagnostic technologies, exploring their capabilities and contributions to the field of TBM research and diagnosis. A comprehensive schematic illustration is provided in Figure 1.

### 2.1. Molecular biology technologies

#### 2.1.1. Polymerase chain reaction (PCR)

Nucleic acid amplification tests (NAATs), such as PCR, hold particular promise for improving TBM diagnosis. The GeneXpert MTB/Rif test is a rapid, automated, cartridge-based nucleic acid amplification test that the World Health Organization (WHO) recommended in 2015 as the initial microbial diagnostic test for TBM (World Health Organization [WHO], 2015). In a recent Cochrane review, the summarized sensitivity of cerebrospinal fluid (CSF) Xpert against CSF culture was 71.1% (95% CI: 62.8–79.1%), and the summarized specificity was 96.9% (95% CI: 95.4–98.0%) (Kohli et al., 2021). Subsequently, GeneXpert MTB/Rif Ultra (Xpert Ultra) was developed (with a larger specimen volume reaching the PCR reaction, additional probes for two other DNA targets, optimized microfluidics, and PCR cycling), featuring enhanced sensitivity and more reliable rifampicin resistance detection (Bahr et al., 2018; Donovan et al., 2020). In 2017, the WHO recommended adopting Xpert Ultra for TBM diagnosis, replacing Xpert as the first-line test (World Health Organization [WHO], 2017).

#### 2.1.2. Analysis of miRNA

Exosomes, which are microvesicles emanating from viable cells into the circulatory system and typically ranging between 30–100 nanometers in diameter, harbor RNA and protein constituents (Wang et al., 2022). Recently, these extracellular vesicles have ascended as potent instruments for the identification of biomarkers in a plethora of diseases, with miRNA identified as one of the most auspicious candidates. A small selection of studies centered on tuberculosis has illuminated the profiles of exosomal miRNAs. Research conducted by Singh et al. (2015) and Alipoor et al. (2017) divulged a differential spectrum of exosomal miRNAs originating from macrophages infected with mycobacterium tuberculosis, implicating the regulatory and diagnostic capabilities of these miRNAs during the infection. Additional studies have also indicated the feasibility of utilizing exosomal miRNAs for differentiating tuberculosis patients from healthy states (Lv et al., 2017).

Further, Hu et al. (2019) discerned six differentially expressed exosomal miRNAs in tuberculosis cases; three of these exhibited substantial discriminatory capacity for TBM and were subject to support vector machine (SVM) modeling. The study also tentatively amalgamated electronic health records (EHR), a digital version of patient medical histories, with miRNA data, proposing the integration of multimodal datasets.

#### 2.1.3. mNGS

Over recent years, mNGS has ascended as a potent sequencing-based modality capable of pathogen identification, without the prior knowledge of the target (Chen et al., 2022). Notably, in contrast to microorganism-specific PCR techniques, mNGS exhibits heightened sensitivity for detecting low-abundance microbial infections in a solitary assay. A seminal pilot investigation involving 12 tunnel boring machine cases revealed a diagnostic sensitivity of 67%, surpassing traditional methods like acid-fast bacilli (AFB) staining, PCR, and microbial culturing (Wang et al., 2019).

In a retrospective analysis of 51 inpatients without HIV suspected TBM, CSF mNGS had a sensitivity of 84.4% (38/45, 95% CI: 69.9–93.0%) and a specificity of 100% (6/6, 95% CI: 51.68-100%) against consensus case definitions (Yan et al., 2020).

### 2.2. Immunological technologies

#### 2.2.1. Interferon-gamma release assays (IGRA)

Interferon-gamma release assays, which are founded on T-cell-based methodologies, serve as diagnostic tools for identifying infections caused by Mycobacterium tuberculosis (Lalvani et al., 2001). Currently, two commercial types of IGRAs are available: T-SPOT. TB (T-SPOT, Oxford Immunotec Ltd., Oxford, UK) and QuantiFERON-TB Gold (QFT, Cellestis Ltd., Carnegie, Australia or Qiagen, Hilden, Germany) (Bastian and Coulter, 2017). These assays employ enzyme-linked immunospot and enzyme-linked immunosorbent assay techniques, respectively (Uplekar et al., 2015). A meta-analysis and systematic review from 2016 determined that the overall sensitivity rates for blood and CSF IGRA were 78 and 77%, accompanied by specificity rates of 61 and 88% (Yu et al., 2016). These data suggest that these assays have only moderate accuracy. Specificity is enhanced when the assays are used on CSF, but large volumes are required (>2 ml) and indeterminate results are common (up to 15%). Consequently, the deployment of IGRA should be augmented by additional diagnostic modalities and comprehensive clinical assessments for a more reliable and precise diagnosis.

### 2.3. Biomarker analysis

#### 2.3.1. Protein and metabolite analysis

The analysis of proteins and metabolites leverages advanced high-throughput technologies to carry out comprehensive and quantitative assessments of low-molecular-weight metabolites in biological specimens. These technologies include proton nuclear magnetic resonance (^1^H-NMR), which is a technique that uses the magnetic properties of atomic nuclei for structural analysis; liquid chromatography-mass spectrometry (LC-MS), a powerful tool combining the separating capabilities of liquid chromatography with the quantitative and qualitative analysis abilities of mass spectrometry; and gas chromatography-mass spectrometry (GC-MS), which is similar to LC-MS but specializes in the analysis of volatile compounds. These metabolite profiles can serve as molecular characteristics of the disease state, offering valuable information for diagnosis, disease progression, and treatment efficacy (Qiu et al., 2023). In the context of TBM, the expression patterns of specific metabolites in cerebrospinal fluid and blood could be closely related to the onset, development, and prognosis of the disease (van Zyl et al., 2020; Gao et al., 2023). Precise analysis of these metabolites not only helps in enhancing the accuracy of early TBM diagnosis but may also reveal its pathophysiological mechanisms and contributing factors (Cao et al., 2022). For example, CSF lactate and CSF glucose, as the two primary metabolic markers identified from CSF metabolomics studies, have already been instrumental in the diagnosis of TBM. Crucially, for the diagnosis of TBM, the observed concentration ranges are 3.04–17 mmol/L for CSF lactate and 1.6–2.69 mmol/L for CSF glucose (Mason and Solomons, 2021). Although metabolomics has huge potential in TBM diagnosis, it also faces the complexity of sample handling, challenges in data analysis, and the need for more clinical validation studies.

### 2.4. Imaging technologies

#### 2.4.1. MRI

As a high-resolution, non-invasive imaging technique, MRI can provide detailed views of the anatomy and physiological activities of the central nervous system, including the brain, brainstem, and spinal cord. In the diagnosis of TBM, MRI is generally used for detecting inflammation in the meninges, ventricles, and brain tissues, including manifestations such as meningeal thickening, brain edema, ventricular dilation, and localized ischemia or hemorrhage (Pienaar et al., 2009). Advanced MRI technologies like diffusion tensor imaging (DTI) and functional magnetic resonance imaging (fMRI) can further assess microstructural changes in neural conduction and brain function (Mathur et al., 2010; Ding et al., 2013). This information is highly valuable for early diagnosis, disease severity assessment, and monitoring treatment responses.

However, the application of MRI also has certain limitations, including high cost, limited availability of equipment, and reliance on patient cooperation. Additionally, the interpretation of MRI results requires specialized skills and experience. Despite this, MRI serves as a powerful diagnostic tool indispensable for accurate TBM diagnosis, treatment planning, and efficacy assessment, and may continue to play a crucial role in future research and clinical practice.

## 3. Fundamentals of machine learning

Applications of machine learning in the realm of biomedical sciences have increasingly captivated scholarly attention. Fundamentally, machine learning methodologies bifurcate into two cardinal classifications: supervised and unsupervised learning (Greener et al., 2022).

Supervised learning is predicated on utilizing expertly annotated datasets to train computational models for the extraction of specific, disease-related attributes. Upon rigorous training, such models acquire the capability to discern and categorize pertinent attributes within novel, unlabeled datasets, thereby augmenting clinical diagnostics (LeCun et al., 2015). From a technical perspective, supervised learning can be subdivided into classification and regression algorithms. Classification algorithms strive to categorize data samples, while regression algorithms aim to predict continuous variables. Specific techniques encompass logistic regression (LR), decision trees (DT), random forests (RF), neural networks (NN), and deep learning. Importantly, the majority of machine learning algorithms feature both classification and regression variants, rendering the choice of algorithm contingent upon the task at hand.

Conversely, unsupervised learning seeks to unearth latent structural or pattern-related nuances in data without the crutch of pre-labeled datasets. This approach shines in its ability to handle complex, high-dimensional data, such as gene expression profiles (Becht et al., 2019). Through unsupervised learning, one can efficaciously identify co-expression modules in genes, potentially indicative of common biological mechanisms or pathways (Tawa et al., 2021). Noteworthy is the emergent interest in semi-supervised learning methods, which amalgamate the virtues of supervised and unsupervised paradigms to bolster classification performance through clustering techniques (Dou et al., 2023).

**FIGURE 2:**
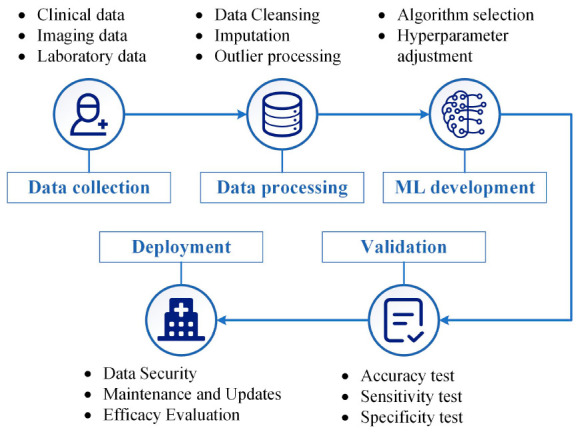
General process for applying machine learning in medical diagnosis and treatment. It outlines crucial steps such as data collection, data processing, machine learning (ML) development, validation, and eventual clinical deployment. The figure aims to offer a roadmap for clinicians and researchers interested in integrating machine learning into medical practice.

The integration of machine learning into medical practice constitutes a multi-stage, interdisciplinary endeavor. Initially, a dataset that is both large and representative is assembled, often comprising medical records and biomedical imaging. The data quality is crucial for model efficacy. Subsequently, meticulous pre-processing eliminates noise and balances the dataset, if necessary. Upon dataset preparation, algorithmic model development commences. Models, ranging from traditional DT to NN, are trained and fine-tuned on a data subset to optimize predictive capabilities. Following training, the model undergoes rigorous validation using an independent dataset and standardized evaluation metrics such as accuracy, sensitivity, and specificity. After successful validation, the model transitions to clinical deployment, serving as an auxiliary tool for clinicians in diagnosis and treatment planning. Continuous maintenance and periodic re-validation are imperative for sustained efficacy. The overall process is encapsulated in Figure 2, providing a roadmap for medical professionals interested in machine learning applications.

## 4. Research status of machine learning in meningitis

As meningitis research grows, more studies are employing diverse methods, evolving from traditional statistics to machine learning models, to diagnose the disease in hospital settings. These studies, especially those using larger datasets and multiple clinical factors, have shown improved predictive accuracy. Advanced diagnostic technology has also expanded the variety of features included in these models. Table 1 summarizes key studies, reviewing aspects like study population, outcomes, features, validation methods, and model performance.

**TABLE 1:** Summary of clinical models for meningitis diagnosis.

Modeling technique	Data type	Number of patients	Model performance	References
LR	CSF features	125	Model Hosmer–Lemeshow goodness-of-fit test: χ2 = 2.486, df = 8, *p* = 0.962.	Wang et al., 2021
LR	CSF features	80	Model AUC was 0.916 (95% CI: 0.857–0.976), sensitivity was 0.95 and specificity was 0.775.	Huang et al., 2022
LR and CART.	Clinical features	205	Model sensitivities were 0.99 (LR) and 0.87 (CART).	Török et al., 2007
LR	Clinical features	382	Model AUC was 0.923, sensitivity was 0.858 and specificity was 0.877.	Lu et al., 2021
LR and CART.	Clinical features	508	Model sensitivities were 0.906 (LR) and 0.91 (CART).	Dendane et al., 2013
LR and CART.	Clinical features	251	Model sensitivities were 0.86 (LR) and 0.88 (CART).	Thwaites et al., 2002
LR	CSF features	174	Model AUC was 0.923 (95% CI: 0.867–0.980).	Luo et al., 2021
LR	Clinical features	167	Model sensitivity, specificity and positive predictive value was 0.471, 0.951 and 0.909.	Handryastuti et al., 2023
Ensemble algorithms (Bagging + NBTree)	Clinical features	26288	Model AUC was 0.95, accuracy, precision, recall and f-measure was 0.89.	Guzman et al., 2022
ANN	Clinical features	1000	Model accuracy was 96.69%	Šeho et al., 2022
ANN	Clinical features	203	Model AUC was 0.85 (95% CI: 0.79–0.89).	Jeong et al., 2021
DT	Clinical features	22602	Model AUC was 0.95, precision was 0.942.	Lélis et al., 2017
DT	Clinical features	26288	Model accuracy was 0.943.	Lelis et al., 2020
SVM	mNGS of CSF	368	Model AUC was 0.88, sensitivity was 0.889 and specificity was 0.88.	Ramachandran et al., 2022
SVM	miRNAs and EHRs	370	Model AUC was 0.97 (95% CI: 0.80–0.99).	Hu et al., 2019
LR and RF	MRI radiological features	216	Model AUC was 0.954, accuracy was 0.909 and F1 score was 0.928.	Aftab et al., 2021
LR and SVM	MRI radiological features	371	Model AUC was 0.796 (95% CI: 0.744–0.847).	Ma et al., 2022

### 4.1. Research based on traditional statistical methods

Earlier diagnostic models for TBM relied on relatively small sample sizes and traditionally employed statistical methods such as logistic regression. Wang et al. (2021) aimed to assess the clinical features associated with normal CSF protein levels in pediatric TBM. Conducted retrospectively, their study specifically examined two clinical features: vomiting and serum glucose levels. The research indicates that these features are correlated with normal CSF protein levels in children with TBM. This finding is particularly significant for diagnosing and managing pediatric TBM, as CSF protein is often employed as a crucial diagnostic marker for the disease. However, the study has several limitations, including a small sample size and the focus on a single research center, which may restrict its broader applicability. The study also did not explore the relationships between CSF protein and other CSF analytes.

Huang et al. (2022) employed ELISA assays to examine the expression of eight proteins in the CSF of 80 patients, which included 22 confirmed cases of TBM, 18 probable cases, and 40 non-TBM cases. They discovered significant differences in the expression of seven proteins between TBM and non-TBM groups. Through unsupervised hierarchical clustering analysis, the researchers further identified a pattern composed of these seven differentially expressed proteins. Logistic regression analyses validated the high efficacy of a combination of three biomarkers (APOE, APOAI, S100A8) in distinguishing TBM from non-TBM cases, with an AUC of 0.916, a sensitivity of 0.95, and a specificity of 0.775.

In contrast, Török et al. (2007) focused on regions with high tuberculosis incidence but limited laboratory resources. They selected a sample of 205 HIV-negative meningitis patients with lower CSF glucose levels. Employing LRand CART, the researchers successfully classified patients into TBM and bacterial meningitis (BM) groups. The LR model achieved a diagnostic sensitivity of 0.99 for TBM and 0.815 for BM, whereas the CART method reached diagnostic sensitivities of 0.87 for TBM and 0.865 for BM. These algorithms primarily relied on factors like age, white blood cell counts in blood and CSF, medical history, and the percentage of neutrophils in the CSF for diagnosis.

Dendane et al. (2013) used data from 508 patients—comprising 274 cases of TBM and 234 cases of bacterial meningitis—to apply logistic regression models and CART analyses. They successfully identified six variables significantly associated with TBM diagnosis. These variables include female gender, symptom duration exceeding 10 days, focal neurological signs, blood white cell count less than 15 × 10^9/L, serum sodium below 130 mmol/L, and a total CSF white cell count less than 400 × 10^6/L. The sensitivity and specificity of this algorithm ranged between 0.87 and 0.88, and between 0.95 and 0.96, respectively.

Thwaites et al. (2002) analyzed data from 251 adult patients in a Vietnamese infectious disease hospital, consisting of 143 TBM cases and 108 bacterial meningitis cases. The researchers pinpointed five features strongly correlated with TBM diagnosis: age, length of illness, white cell count, total CSF white cell count, and the proportion of neutrophils in the CSF. Based on these key features, the team formulated a diagnostic rule and evaluated it through both retrospective and prospective test data methodologies. The diagnostic rule demonstrated 0.97 sensitivity and 0.91 specificity in retrospective testing, and 0.86 sensitivity and 0.79 specificity in prospective testing.

Luo et al. (2021) constructed a diagnostic model based on multiple CSF markers and the TBAg/PHA ratio. Through multivariate logistic regression analysis, the model incorporated four key variables: CSF chloride levels, CSF nucleated cell count, the proportion of lymphocytes in CSF, and the TBAg/PHA ratio. The model achieved a sensitivity of 0.8158 and a specificity of 0.9184, with an accuracy exceeding 0.85 and an area AUC of 0.949.

While these diagnostic models are high-performing, they often function as “black boxes,” offering limited interpretability for clinicians. In contrast, clinical scoring tools are generally more accessible to healthcare professionals, being based on clear, intuitive variables and scoring systems. To address this, Lu et al. (2021) introduced a comprehensive new diagnostic scoring system, which synthesizes 28 clinical, laboratory, and radiological factors to differentiate TBM from other common central nervous system infections. This system, validated in a prospective cohort, excelled in sensitivity and specificity, achieving 0.858 and 0.877, respectively.

Similarly, Handryastuti et al. (2023) developed a simplified scoring system for diagnosing pediatric TBM based on a retrospective analysis and multivariable prediction model. Although the system has lower sensitivity at the established threshold, reaching 0.471, its high specificity of 0.951 signifies a robust accuracy in clinical diagnosis.

### 4.2. Research based on machine learning methods

In light of increasing dataset sizes, several studies have been published over the past few years that employ machine learning methods with larger data set requirements, such as SVM and tree-based models. Guzman et al. (2022) scrutinized a substantial dataset consisting of 26,228 patients, characterized by 19 primary variables related to symptoms and initial CSF laboratory results. The central aim of their research was to identify the most effective classifier for meningitis etiology. Toward this goal, they explored a myriad of feature selection, dataset sampling, and classification model techniques, based predominantly on ensemble methods and DT. Following experimentation with 27 classification models, 19 of which employed ensemble methods, they found that the ensemble methods yielded the most optimal classifiers. Specifically, the union of Bagging and naïve bayes trees (NBTrees) resulted in peak performance metrics, boasting an F-measure of 0.89, along with an accuracy, recall, and AUC of 0.95 each. Their study also illustrated that, compared to using DT alone, the incorporation of ensemble methods substantially enhanced the model’s diagnostic efficacy.

Šeho et al. (2022) deployed a dataset of 1,000 instances, where 800 were meningitis patients and 200 were healthy individuals. Factors used in diagnosing meningitis included body temperature, protein levels, CSF-to-blood glucose ratio, CSF white cell counts, lactate, glucose, erythrocyte sedimentation rate, and C-reactive protein (CRP). They developed a classifier that utilized ANN for instance categorization. When tested on the employed dataset, the proposed system exhibited a classification accuracy of 0.9669.

Jeong et al. (2021) applied a range of machine learning models, including Naive Bayes, LR, RF, SVM, and ANN, to differentiate between TBM and viral meningitis (VM). The study cohort consisted of 203 patients, incorporating data from 143 confirmed cases of VM and 60 cases of confirmed or probable TBM. Among all tested machine learning techniques, ANNs using imperative estimators yielded the highest AUC registering at 0.85 with a 95% confidence interval ranging from 0.79 to 0.89.

Lélis et al. (2017) compiled a dataset of 22,602 potential meningitis cases in Brazil. Utilizing input data from nine symptom categories, alongside other patient information like age, gender, and location, they applied seven classification techniques and validated their models using 10-fold cross-validation. Their results indicated that the deployed methods could appropriately diagnose pneumococcal meningitis.

Further extending the scope, Lelis et al. (2020) developed an integrated clinical decision support system (CDSS) aimed at assisting physicians in making early stage meningitis diagnoses based on observable symptoms. Built on explainable, tree-based machine learning models and knowledge engineering techniques, this system integrated three intelligent components. The system was constructed and assessed on a Brazilian dataset encompassing 26,228 meningitis patients and demonstrated exemplary classification performance, particularly for the more severe type of meningitis, termed as MD-type, with an accuracy rate as high as 94.3%. Further experimentation corroborated that the system could accurately diagnose 88% of meningitis cases in a real-world database. This research holds particular importance for regions lacking financial resources and advanced medical facilities, as it offers an accurate, economical, and actionable methodology for early stage diagnosis.

In recent years, novel multimodal data types such as metagenomic sequencing of CSF, exosomal miRNAs, and electronic health records have enriched the data resources available for machine learning models. This data diversity enables models to learn from multiple perspectives, thereby augmenting their diagnostic and predictive capabilities.

Ramachandran et al. (2022) sought to enhance the diagnostic accuracy of TBM and its mimic diseases through an integrated machine learning classifier that combines metagenomic sequencing of CSF and host gene expression. Conducted in the sub-Saharan African region—a zone where TBM is prevalent yet challenging to diagnose—the study employed methods including the extraction of total nucleic acids from CSF samples followed by RNA and DNA sequencing, which were then analyzed using a machine learning classifier. Overall, the study found that this combined approach demonstrated high sensitivity and specificity in diagnosing TBM. In the test set, the diagnostic accuracy was 0.88, with sensitivity and specificity rates at 88.9 and 88%, respectively. Notably, the study also showed that this approach performs reliably in resource-constrained settings.

Hu et al. (2019) employed a combination of exosomal miRNA and electronic health data to conduct diagnostic studies on tuberculosis among 351 individuals, which included both active tuberculosis patients and a control group. The authors utilized an ExoQuick Kit and thrombin D to isolate exosomes from plasma samples, which were subsequently validated through nanoparticle tracking analysis, transmission electron microscopy, and protein blotting. In the exploratory phase, 102 exosomal miRNAs exhibited differential expression between tuberculosis patients and the healthy control group. Ten of these differentially expressed exosomal miRNAs were selected for further analysis. This study not only introduced new biomarkers but also optimized existing diagnostic methods, attaining an AUC of 0.97 (95% CI: 0.80–0.99) in diagnosing TBM.

With the emergence of imaging genomics, an increasing number of investigators are harnessing the power of machine learning algorithms in conjunction with a diverse array of radiological features derived from MRI scans to enhance the diagnostic precision of brain tuberculosis.

Aftab et al. (2021) utilized patient data from Aga Khan University Hospital in Pakistan, encompassing not only demographic information but also radiological features derived from MRI. To address class imbalance during data preprocessing, the study employed various oversampling techniques for the minority class, such as SMOTE, SMOTE-TOMEK, SMOTE-ENN, and ADASYN. Two primary classification models, LR and RF, were tested. The LR model in combination with SMOTE + TOMEK techniques yielded the highest diagnostic performance, achieving an accuracy of 90.9%, an AUC of 95.4%, and an F1 score of 92.8%. These findings underscore the significant accuracy and effectiveness of this machine learning approach in the diagnosis of brain tuberculosis, particularly in emergency or clinical settings where rapid and accurate diagnosis is imperative.

Ma et al. (2022) developed an automated, non-invasive diagnostic tool for early detection of basal cistern changes in TBM using deep learning and radiomics methods on a multi-center MRI dataset. The authors initially employed an nnU-Net-based model for the automatic segmentation of the basal cistern region, achieving an average dice coefficient of 0.727. Subsequently, radiomics features were extracted from FLAIR and T2W images and subjected to independent sample *t*-tests and pearson correlation coefficient analyses for feature selection. Finally, radiomics signatures were constructed using SVM and LR, and their performance was evaluated using receiver operating characteristic (ROC) curves, calibration curves, and decision curve analysis (DCA). Test results indicated that the AUCs for SVM classifiers based on T2W and FLAIR features were 0.751 and 0.676, respectively, signifying good discriminative ability. This integrated method demonstrated considerable potential in the early identification of subtle basal cistern changes in TBM, promising improvements in the early diagnosis and treatment of the disease.

## 5. Discussion

**FIGURE 3:**
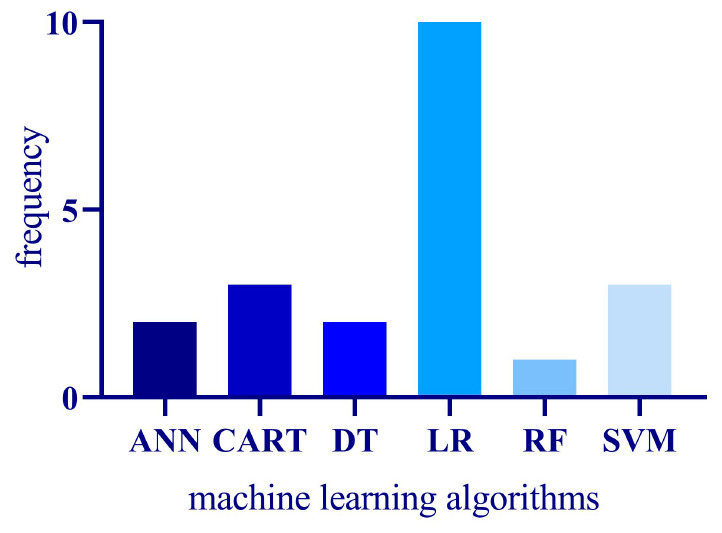
The frequency of modeling techniques employed for diagnosing TBM.

As illustrated in Figure 3, logistic regression emerges as the most favored statistical approach, extensively adopted in the domain of TBM research. In contrast, machine learning algorithms such as SVM and tree models are the second most frequently utilized methodologies for model construction. Notably, a marked uptick in research articles using machine learning for model creation has been observed since 2022, while studies relying on traditional statistical approaches like logistic regression were predominantly concentrated prior to 2021. This trend may signal the growing acceptance and proliferation of machine learning in the field.

Given its data-centric nature, machine learning offers significant potential in the diagnosis of TBM, especially in the last decade, where advancements in novel diagnostic technologies have supplied a variety of data types. As shown in Table 2, these include genomic data, transcriptomic data, fluorescent markers, metabolomic data, proteomic data, radiographic images, and standard clinical and EHR. Such diverse data types not only enhance diagnostic accuracy but also serve as rich resources for training and validating machine learning models. Specifically, the amalgamation of these data types with advanced machine learning and deep learning techniques could pave the way for innovative diagnostic pathways. Some potential applications are outlined below.

**TABLE 2:** Summary of available data type from new diagnostic technologies.

New diagnostic technologies	Available data type
Molecular biology technologies	Genomic data, transcriptome data
Immunological technologies	Fluorescent labeling information
Biomarker analysis	Metabolome data, protein data
Imaging technologies	Radiographic image
Other traditional technologies	Routine clinical data, laboratory testing and EHR.

### 5.1. Molecular biology data

Deep learning algorithms such as convolutional neural network (CNN) and recurrent neural network (RNN) have the capacity to process high-dimensional and complex data structures. This enables them to accurately identify genes and transcription factors associated with TBM from extensive genomic or transcriptomic data. Compared to traditional machine learning and statistical methods, these deep learning algorithms are better equipped to handle data of higher dimensions and complexity, making them more suitable for identifying key elements within intricate biological networks and pathways (Zou et al., 2019; Kleppe et al., 2021; Wang et al., 2023). For instance, deep learning has already been successfully employed in cancer diagnostics to analyze transcriptomic data for the identification of specific gene expression patterns related to certain types of cancer (Coudray et al., 2018; He et al., 2020; Meng et al., 2023). This approach could be similarly applied to the study of TBM, wherein deep learning-based analyses of gene expression data could potentially unveil underlying biological changes in TBM patients, thus aiding in diagnosis and treatment.

### 5.2. Immunological data

The utilization of immunological data offers significant prospects in researches of TBM. High-dimensional immunological datasets can be adeptly navigated using unsupervised and semi-supervised algorithms, such as k-means clustering and autoencoders (Tanner et al., 2013). These computational techniques not only enable the recognition of TBM-associated immune response configurations but also disclose potentially determinative immunological markers and subpopulations of cells that are impactful in the course and responsiveness of treatments. Through the application of high-throughput methodologies like immunohistochemistry and flow cytometry, scholars have effectively pinpointed specific subpopulations of immune cells that correlate with prognostic and therapeutic outcomes (Zhang Z. et al., 2019; Kim et al., 2021; Ye et al., 2022). In a parallel vein, the aggregate analysis of TBM immunological data through similar unsupervised and semi-supervised machine learning algorithms may unearth key immunological metrics pertinent to the dynamics of disease and treatment. Such advanced analytical processes could contribute to the refinement of diagnostic frameworks and could potentially catalyze the formulation of more individualized treatment strategies.

### 5.3. Biomarker data

The value of metabolomics in the diagnosis of TBM has drawn the attention of researchers. Reduced glucose concentrations and elevated levels of proteins in the CSF have long been the two biochemical indicators used to diagnose TBM (Zhang et al., 2018; Zhang P. et al., 2019). However, a lot of information hidden in high dimensional data is often overlooked. To extract valuable information from these high-dimensional and variable biomarker data sets, machine learning algorithms like RF and SVM could be a solution (Reel et al., 2021; Sen et al., 2021; Sun et al., 2022). These algorithms exhibit exceptional feature selection and classification capabilities, enabling the identification of key metabolites and protein markers correlated with the diagnosis and prognosis of TBM.

### 5.4. Radiological imaging

The past few years have seen research initiatives that employ radiomics to manually extract features related to TBM (Aftab et al., 2021; Ma et al., 2022). However, the advent of CNN offers an automated, end-to-end analytical approach that achieves accuracy levels that meet or even surpass those of human experts (Hosny et al., 2018). For example, Jang et al. (2018) exemplified that CNN could autonomously identify glioblastoma features in MRI scans with a remarkable 0.87 AUC, significantly outperforming traditional image analysis methods. Therefore, employing this end-to-end approach to the analysis of radiological images may be helpful in the diagnosis of TBM. Not only can complex biomarkers be automatically identified and analyzed, but more personalized treatment options are also possible. Such a fusion is expected to dramatically improve the accuracy of early diagnosis and disease surveillance, thereby optimizing treatment outcomes and improving patient quality of life.

### 5.5. Routine clinical data and EHR

In the realm of healthcare informatics, routine clinical data along with EHRs play an indispensable role. These expansive, multifaceted datasets typically include a wide range of information from clinical narratives and laboratory outcomes to imaging data and individual patient histories. While traditional methods of mining and analyzing these datasets have been laborious and error-prone, requiring manual scrutiny and specialized expertise, recent breakthroughs in natural language processing (NLP) and time-series analytics have revolutionized the process (Esteva et al., 2019). For example, Kehl et al. (2019) demonstrated the successful application of NLP techniques like text classification and entity recognition for the automatic extraction of oncologic outcomes from EHRs. Moreover, the application of time-series analysis has yielded valuable insights for evidence-based clinical decisions, especially in monitoring patient health and forecasting disease trajectories. When applied to TBM (Malo et al., 2021), this method enables the real-time monitoring of patient vitals, pharmacological responses, and disease advancement, thereby facilitating more individualized and prompt healthcare interventions.

## 6. Limitations of the application of machine learning in TBM

Although the past decade has seen increasing research on machine learning-based diagnostic tools for TBM, the field remains relatively nascent. Applying machine learning to the diagnosis of TBM presents several challenges, encompassing data collection, model training, diagnostic accuracy, and practical clinical implementation. The majority of predictive models have been validated using retrospective data, but only a few have been trained and tested on prospective data sets. Another crucial issue is the clinical heterogeneity of TBM, which manifests in symptoms ranging from mild headaches to severe neurological damage (Garg, 2010). This complexity complicates the task of machine learning models in capturing a comprehensive array of variables and clinical presentations that influence disease progression. Consequently, high-quality, and reliable machine learning models necessitate validation through large-scale, multi-center data that span different healthcare systems. Such validation not only enhances model accuracy but also deepens our understanding of the mechanisms of TBM and various prognostic factors. Additionally, data heterogeneity arising from different regions, hospitals, or equipment can introduce variations that may affect the model’s generalizability. However, the scarcity of such multi-center data, combined with the inherent data heterogeneity, currently limits the model’s generalizability and precision. Lastly, it’s worth noting that many emerging machine learning-based TBM diagnostic tools and algorithms are patented or commercialized, resulting in opacity regarding their specific algorithms and datasets. This lack of transparency hampers comprehensive and rigorous evaluations, further impeding advancements in diagnostic accuracy research.

## 7. Conclusion

This review provides a thorough overview of state-of-the-art diagnostic technologies and machine learning methodologies for the diagnosis of TBM. Our principal aim is to encapsulate extant research and scrutinize its application in the domain of TBM. For the purpose of this review, we bifurcate the research landscape into two primary categories: machine learning approaches and classical statistical methods. In the domain of machine learning, the literature is further segmented into categories of supervised and unsupervised learning, featuring key algorithms like SVM, linear discriminant analysis (LDA), k-nearest neighbors (KNN), ANN, boosting algorithms, RF, and k-means clustering. Conversely, traditional statistical methods mainly involve linear regression, logistic regression, chi-square testing, and CART. Overall, SVM is identified as the most widely applied machine learning tool for TBM diagnosis, whereas logistic regression remains the statistical method of choice. In recent years, machine learning has showcased enormous potential for elevating the diagnosis and treatment of TBM, outclassing traditional methods by excelling in the analysis of intricate biomedical data, including genomic sequencing, metabolomics, and proteomics. Future studies could explore integrating various machine learning algorithms into robust ensembled models and empirically validate these against human benchmarks in controlled trials. Additionally, assessing the integration of traditional diagnostics like MRI with emerging machine learning techniques offers a promising avenue for a more holistic diagnostic approach. Such advancements could significantly improve both TBM diagnosis and treatment, ultimately enhancing patient outcomes.

## Author contributions

YS: Writing – original draft, Writing – review and editing. CZ: Writing – original draft, Writing – review and editing. SP: Writing – original draft, Writing – review and editing. YC: Writing – original draft, Writing – review and editing. XM: Writing – review and editing. Writing – original draft. GH: Writing – review and editing. YW: Writing – review and editing, Writing – original draft. HY: Writing – review and editing. CW: Writing – review and editing. HZ: Writing – review and editing, Writing – original draft. WZ: Writing – review and editing. XY: Writing – review and editing. CL: Writing – review and editing. DC: Writing – review and editing. LH: Writing – review and editing. FS: Writing – original draft, Writing – review and editing.
