# Supplementary material for: The diagnosis of tuberculous meningitis: advancements in new technologies and machine learning algorithms
Source: Front Microbiol. 2023 Oct 24;14:1290746. doi: 10.3389/fmicb.2023.1290746 (PMC10628659; doi:10.3389/fmicb.2023.1290746)
Supplement: Supplementary file 1 [file Table_1.DOCX]

**Supplementary material: literature search strategy for systematic review**

**1.Objective**

The objective of this literature search strategy is to identify studies that analyze the application of machine learning (ML) and deep learning (DL) techniques in the diagnosis of tuberculosis meningitis.

**2.Databases and search engines**

PubMed

Google Scholar

**3.Date of search**

The initial search was conducted in July 2023.

**4.Language restrictions**

Only articles written in English were considered for inclusion.

**5.Keywords**

tuberculosis meningitis

machine learning

deep learning

These keywords were used in various combinations and were combined using the 'AND' and 'OR' operators.

**6.Additional identification**

After the initial database search and screening, additional articles were identified by reviewing the cited references in the studies that met our inclusion criteria. This snowballing method helped to ensure a comprehensive review and to capture any relevant studies that might have been missed during the initial database search.
